# Invariant object recognition is a personalized selection of invariant features in humans, not simply explained by hierarchical feed-forward vision models

**DOI:** 10.1038/s41598-017-13756-8

**Published:** 2017-10-31

**Authors:** Hamid Karimi-Rouzbahani, Nasour Bagheri, Reza Ebrahimpour

**Affiliations:** 1 Department of Electrical Engineering, Shahid Rajaee Teacher Training University, Tehran, Iran; 2 Cognitive Science Research lab., Department of Computer Engineering, Shahid Rajaee Teacher Training University, Tehran, Iran; 3 School of Cognitive Sciences, Institute for Research in Fundamental Sciences (IPM), Tehran, Iran

## Abstract

One key ability of human brain is invariant object recognition, which refers to rapid and accurate recognition of objects in the presence of variations such as size, rotation and position. Despite decades of research into the topic, it remains unknown how the brain constructs invariant representations of objects. Providing brain-plausible object representations and reaching human-level accuracy in recognition, hierarchical models of human vision have suggested that, human brain implements similar feed-forward operations to obtain invariant representations. However, conducting two psychophysical object recognition experiments on humans with systematically controlled variations of objects, we observed that humans relied on specific (diagnostic) object regions for accurate recognition which remained relatively consistent (invariant) across variations; but feed-forward feature-extraction models selected view-specific (non-invariant) features across variations. This suggests that models can develop different strategies, but reach human-level recognition performance. Moreover, human individuals largely disagreed on their diagnostic features and flexibly shifted their feature extraction strategy from view-invariant to view-specific when objects became more similar. This implies that, even in rapid object recognition, rather than a set of feed-forward mechanisms which extract diagnostic features from objects in a hard-wired fashion, the bottom-up visual pathways receive, through top-down connections, task-related information possibly processed in prefrontal cortex.

## Introduction

Human object recognition is remarkably rapid and precise. Although many brain-plausible and computer vision algorithms have approached human recognition performance in recent years^1–3^, humans still outperform most of these algorithms in difficult situations of object recognition such as when objects appear under variations^4^ (e.g. size, position, rotation) or when they are occluded^5^ or in clutter^4^. These observations suggest that humans seem to use a set of cognitive processes which are still missing from the computational algorithms and even from brain-plausible models of human vision.

The ventral visual stream of the brain, which starts from V1 and ends at inferior temporal (IT) cortex, is proposed to play the key role in the processing of object category information^6,7^. Along this stream, as the simple to complex visual features are extracted from visual signals, category information reveals increased robustness to different variations^8–12^. Finally, these category-specific representations are classified by high-level cognitive processes in prefrontal cortex^7,13–16^. Accordingly, although this insight implies that a purely hard-wired feed-forward (bottom-up) account might be able to explain invariant object recognition in humans, many studies have revealed a more complex interaction between the bottom-up sensory information and top-down cognitive processes which together determine the performance of object recognition in humans^17–19^. Therefore, as opposed to most of recently developed computational algorithms which have merely implemented the hierarchical feed-forward structure of the ventral visual stream^3,20,21^, brain seems to deploy complementary mechanisms to perform accurately in difficult object recognition situations^22,23^.

A seminal study has recently proposed that humans rely significantly on specific sets of object parts (i.e. visual features or simply features), referred to as Minimal Recognizable Configurations (MIRCs), while none of the state-of-the-art computational algorithms (i.e. including machine-vision algorithms^24–26^ as well as brain-inspired models of vision^8,20,27^) revealed such a dependence on specific object parts in recognition^28^. The MIRCs were called minimal in the sense that a small reduction in their complexity caused a significant reduction in humans’ recognition performance (but did not affect performance of the models). In other words, some specific object parts were considered more informative to humans, but provided as much information as any other parts for computational models. That study provided proof for a huge gap in object processing between humans and computational algorithms of object recognition. However, this should not come as a surprise that computational algorithms failed to reflect human recognition strategies, knowing that feature-based object/scene recognition strategies adopted by humans are not even observed in rhesus monkeys^29,30^, the major animal model of human perception.

Altogether, humans’ remarkable recognition performance and their unique way of exploiting object features motivated us to systematically study humans’ feature-based object recognition strategies under variations. It has been proposed that, the redundancy of objects’ diagnostic features can explain how invariance is achieved in recognition^28^. In other words, variations would impose little impact on recognition as long as there are other diagnostic features available to the visual system from a given object^28^. However, this suggestion had remained overlooked before the current study.

In this study, we aim to answer two major questions. First, what is the feature-based strategy used by humans when recognizing objects under variations? It can also be similar/different across human individuals. Second, do hierarchically organized feature extractor models of vision adopt the same strategy as humans do for invariant object recognition? By the second question, we aim to know whether feature-based recognition can be simply explained by feed-forward feature extractor mechanisms or other mechanisms might also contribute to invariant object recognition.

Although there are many studies which have investigated feature-based recognition in humans and other species^31–37^, no systematic study has ever been conducted to investigate this ability under controlled naturalistic variations. In a comparative study between humans and monkeys, it was shown that humans relied on sets of relatively consistent (invariant) features when objects were rotated in image plane, whereas monkeys used a screen-centered (view-specific) strategy^29^. However, the simplicity of the task (i.e. subjects discriminated three semantically and perceptually different objects which were presented for 500 ms) and stimuli (i.e. stimuli were dark silhouettes of bottle, pipe and hand), made the results inconclusive^29^. Moreover, that study did not cover more challenging variations such as size, position and in-depth rotation, each of which may involve different mechanisms in the human brain^6,38^. A recent study on rats incorporated all the mentioned variations and showed that rats followed a relatively invariant feature-based strategy when recognizing objects^36^. A follow-up study on rats showed that the consistency (invariance) of the diagnostic features was directly related to the level of similarity between the objects which were discriminated^37^. The former study also showed that a pixel-space ideal observer could accurately predict rats’ discrimination strategy, suggesting that rats might have developed a template-matching pixel-space strategy for object recognition. However, it remains unknown what strategies would humans adopt to perform a similar task.

Therefore, to extend those previous results to the human feature-based recognition, we generated an image set which presented objects in thirteen different conditions in four variations. We then asked humans to discriminate a pair of 3D objects in two psychophysical object discrimination tasks. In order to obtain the diagnostic and anti-diagnostic object parts (features), Bubbles method was used in a staircase paradigm^39^. To provide computational cases for comparison, we also investigated the strategies used by a pixel-level ideal observer^39^ and a deep convolutional neural network^20^ (i.e. known as AlexNet) by feeding them the same image set provided to humans. Using the ideal observer enabled us to compare human results with those previously reported for rats^36,37^. We used AlexNet as a model of human vision, as it was suggested to provide the most brain-plausible object representations to humans and monkeys^2,21,40^.

We show that when discriminating the naturalistic images of this study, humans relied on a few object features (diagnostic features) in each variation condition which mostly varied from subject to subject. These diagnostic features could be relatively consistent (invariant) across variation conditions, supporting an invariant rather than screen-centered feature-based recognition strategy adopted by humans. Interestingly, the level of diagnostic features consistency was determined by the level of similarity between the two objects which were discriminated. We also show that, neither an ideal observer nor a deep convolutional neural network could emulate human recognition strategies; those models developed screen-centered strategies. This implies that, compared to humans, who can generalize the diagnostic features from one variation condition to another, the hierarchical models of vision used in this study, adopt a unique strategy for each variation condition. As these computational models present low to high levels of feature extraction mechanisms in the hierarchy of the human visual system, these results suggest that human object recognition involves more than just a series of feature extraction levels; it can rather be highly influenced by top-down cognitive mechanisms such as task-information, learning and decision-making.

## Methods

### Image set generation

**Figure 1:**
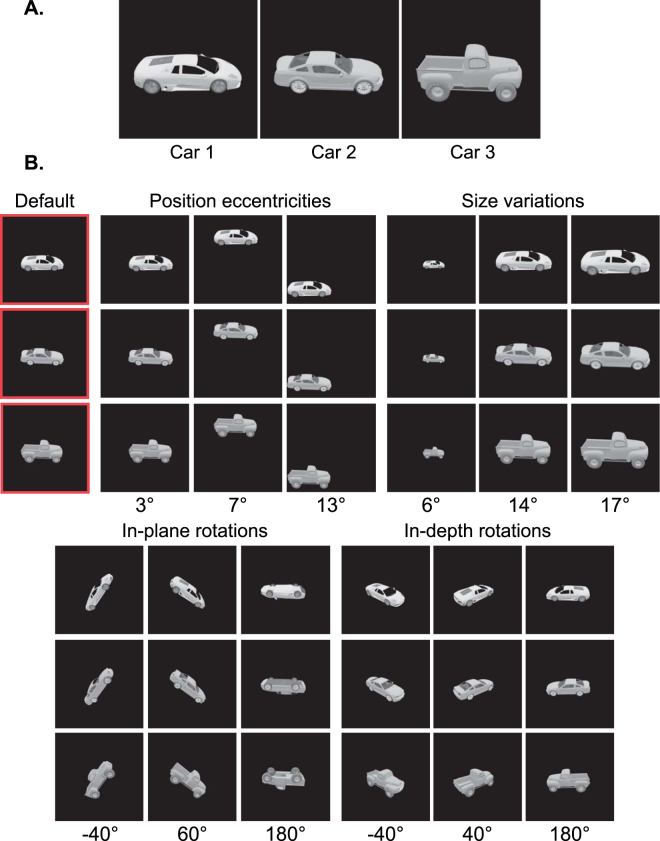
Image set. (**A**) Car models used in the behavioral and simulation experiments. (**B**) Variation conditions that the cars underwent, which included variations in position, size, in-plane and in-depth rotations. In position conditions, cars appeared on imaginary circles with different radii from the image center. In size conditions, they appeared at different scales and in the in-depth and in-plane conditions, cars were rotated around Z and Y Cartesian axes, respectively. Note that, images were cropped and rescaled in different variations for better visualization in the images shown. The default condition, which shows the cars in 10° size, zero eccentricity, zero in-plane and in-depth rotations, is indicated by the red frame. More information regarding each condition is provided below it. The 3D car models used to generate these images are available under a Creative Commons Attribution-ShareAlike 3.0 Unported License (https://creativecommons.org/licenses/by-sa/3.0/) and were freely downloaded from (https://grey.colorado.edu/CompCogNeuro/index.php/CU3D).

To study feature-based invariant object recognition in humans and computational models, we needed an object image set with levels of controlled variations. The number of objects in the image set was limited by the Bubbles method and our goal to study several conditions in four different variations. Thus, three 3D car mesh models were freely downloaded (available at https://grey.colorado.edu/CompCogNeuro/index.php/CU3D) and rendered in Blender software (www.blender.com) in different variation conditions. Two of the car models were sport cars (cars 1 and 2) and the third (car 3) was a truck. The car models were illuminated from the same light source (Fig. 1A). They covered approximately equal areas and had equal average luminance. In the default condition, cars were positioned in the middle of the screen (no eccentricity), covered an average area of 10 degrees of visual angle and underwent no in-plane and in-depth rotations. In order to generate different variation conditions, cars underwent levels of variation in position, size, in-plane and in-depth rotation (Fig. 1B). Variation levels were chosen so as to provide overlapping and non-overlapping viewing presentations across conditions. This way, the conditions could provide a large range of performance when humans tried to recognize the objects (e.g. in very small-sized or upside down views). Finally, a unique 768 × 768 pixel gray scale (i.e. ranging from 0 to 255 of gray levels) image was generated from each car in each variation condition making to a total of 39 unique images in the unmasked image set for the three cars (i.e. each car underwent 13 variation conditions).

**Figure 2:**
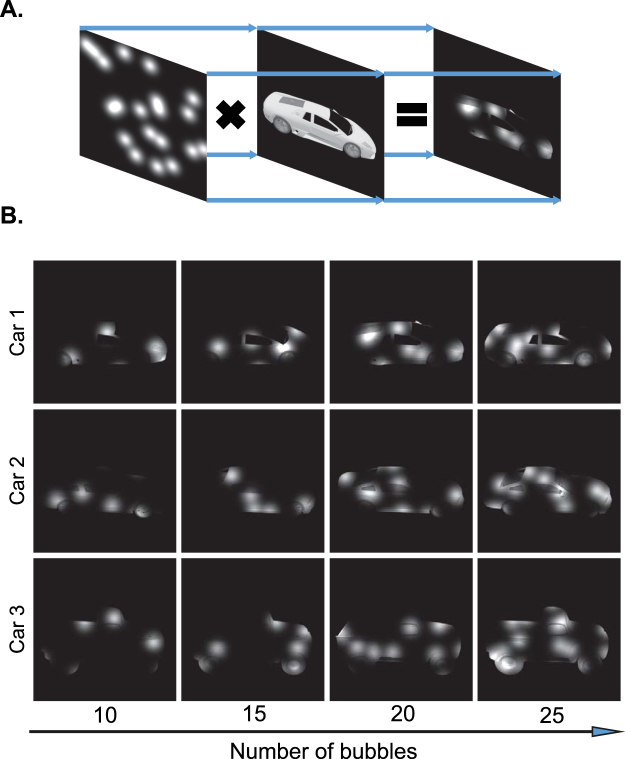
Bubbles procedure. (**A**) Object image is multiplied by bubbles masks with random Gaussian pores at different positions to generate the final masked image. (**B**) Different numbers of bubbles, as indicated by the number below each image, were used in the masks to control the discrimination performance in the behavioral experiment. The 3D car models used to generate these images are available under a Creative Commons Attribution-ShareAlike 3.0 Unported License (https://creativecommons.org/licenses/by-sa/3.0/) and were freely downloaded from (https://grey.colorado.edu/CompCogNeuro/index.php/CU3D).

In order to obtain the visual features which were most relevant to the recognition of each object, we used Bubbles method^39^. This method, previously used in several object recognition tasks on humans and other species^32,36,37,39,40^, assigns weights to different object patches (features) according to their contribution to correct recognition of the corresponding object. To find the contributing features, a bubbles mask is put on the object image which allows only a fraction of the object to be recognizable by the observer (Fig. 2A). The bubbles mask used in this study was a gray scale image with gray level values between zero and one. These masks were generated by adding several normally-distributed gray level values at random 2D positions on a totally zero mask. After multiplying the object image by the generated mask image, the object is only partially observable through the pores.

The recognition performance of observers is highly dependent on both the number and the variance of the Gaussian pores of the bubbles mask. Therefore, to control the recognition accuracy, as it is needed in the analysis of Bubbles method, we chose a constant variance for the pores relative to the object size and controlled the number of pores on the mask (Fig. 2B). In fact, we dynamically altered the number of bubbles on the masks in the range of 10 to 25 in steps of five, based on the subject’s performance using an online adaptive algorithm to keep the accuracy at approximately 75% correct for each subject (Fig. 2B). During each experiment session, this algorithm evaluated subject’s performance on the last four trials to decide on the number of bubbles in the coming trial. Accordingly, the number of bubbles was increased by five if the masked stimuli had been correctly recognized in less than three trials and was decreased by five if they had been correctly classified in the last four trials. The Bubbles on the masks (i.e. Gaussian pores) had a variance of 750 pixels in the default condition which was multiplied by the scale factors of the objects in other object scales. Each image in the image set was then multiplied by 300 distinct random bubbles masks to make a total of 7800 images (26 images for two objects × 300 masks) shown to each subject. It should be noted that, not all transparent pores were positioned distinctly on the mask, nor did they all align with object parts when multiplied by object images. There were cases in which the pores failed to overlap with any object part so leaving no effect.

### Psychophysical experiments

**Figure 3:**
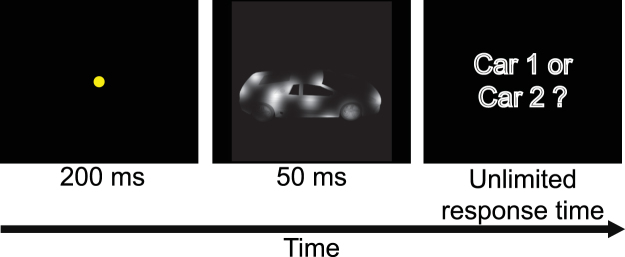
Psychophysical paradigm. Every trial started with a central yellow fixation point which was aimed to preclude subjects from making eye movements, followed by presentation of a masked stimulus, and finished after subject’s response. The 3D car models used to generate these images are available under a Creative Commons Attribution-ShareAlike 3.0 Unported License (https://creativecommons.org/licenses/by-sa/3.0/) and were freely downloaded from (https://grey.colorado.edu/CompCogNeuro/index.php/CU3D).

In order to obtain insight into the underlying mechanisms of feature-based object recognition in humans we conducted two psychophysical (behavioral) experiments. The only difference between the first and the second experiment was the object pairs used in the experiments. It was important for us to study the effect of object similarity on human recognition strategies, as it has been shown to be an effective factor on rats^36,37^. Two distinct groups of 13 and nine human subjects (mean age 26, 15 males) volunteered in two experiments, each of which had 10 sessions of 30-minute task. Informed consent was obtained from each participant. All experimental protocols were approved by the ethical committee of Shahid Rajaee Teacher Training University. All experiments were carried out in accordance with the guidelines of the declaration of Helsinki and the ethical committee of Shahid Rajaee Teacher Training University. The experiments were two-object discrimination tasks. In the first experiment, the first subject group discriminated car 1 from car 2 and in the second experiment the second group discriminated car 2 from car 3. Subjects had normal or corrected-to-normal vision and were seated in a darkened room, 60 cm away from a monitor. Objects were presented in sizes between six to 17 degrees of visual angle and in eccentricities between three to 13 degrees of visual angle, depending on the condition. We used Matlab PsychoToolbox^41^ for stimulus presentation and response recording. Each experiment had two phases: a training phase and a testing phase. The training phase was aimed at acquainting subjects with the stimuli and the mapping between the two objects and the two predefined keys on the keyboard. In the training phase, subjects discriminated all unmasked images of the two objects (i.e. 13 conditions for each object). After these 26 trials, the training phase continued with repetitions of the 26 unmasked images of objects and was stopped as soon as the categorization accuracy, which was being measured from the beginning of the training phase, surpassed 80% correct. At the end of each trial, the correctness of the subject’s response was indicated on the monitor as a feedback to the subject. The order of the presented images was randomized among subjects in both the training as well as the testing phases. As opposed to previous studies in which subjects were tested on viewing conditions (e.g. poses, sizes and positions) which were different from those presented during training^42^, we trained the subjects on all testing conditions to minimize the bias from involving high-level memory mechanisms such as view-point generalization and learning^42^. The testing phase had three differences with the training phase: no feedback was provided to subjects on correctness of their responses; masked versions of object images were presented to subjects; it was organized in four blocks of 195 trials with 5 minutes of rest time in between the blocks. At the beginning of each trial, a fixation point was presented on the center of the screen for 200 ms, followed by a stimulus image which remained on the screen for 50 ms (Fig. 3). After the stimulus offset, although they were asked to respond as fast and accurately as possible, subjects had an unlimited time to determine which car they recognized by pressing the key associated with the car (i.e. the task was two-alternative forced choice).

### Bubbles method

Bubbles method can determine the importance of different object parts in recognition based on correct and incorrect responses of subjects. For that purpose, in the case of HSD experiment, the bubbles masks from trials in which a given subject correctly recognized car 1 were added up in a pixel-wise fashion to generate a ‘correct map’. The whole set of masks, which were used in the first experiment for car 1, was also added up to generate a ‘total map’. The ‘correct saliency map’, which showed the importance of different parts of car 1 in recognition, was calculated by dividing the value of each pixel in the correct map by the pixel value on the total map at the same XY image location.

We conducted a permutation test to find significantly diagnostic regions on the correct saliency map for each object condition, as previously done for rats^36^. For that purpose, the entire set of trials on a single car condition was randomly sub-sampled for a subset of randomly chosen correct trials. The bubbles masks corresponding to the subset were added up and divided by the pooled masks from the whole set of trials to provide a ‘random correct saliency map’. In fact, this map was generated from the bubbles masks including both correct and incorrect trials. These random correct saliency maps included the same number of correct masks as in the actual experiment. This random sub-sampling procedure was repeated 1000 times to reach 1000 random correct maps. Next, a one-tailed permutation test was performed which indicated the significance of each pixel on the true correct saliency map by comparing its pixels with those obtained from the 1000 randomly generated correct saliency maps. Pixels on the true correct saliency maps with values higher the 95^th^ percentile of the corresponding pixel values on the random correct saliency maps were considered significant (p < 0.05) and called ‘diagnostic’ as they provided a high chance for the object to be correctly recognized in the experiment. To find the ‘anti-diagnostic’ pixels (features or regions) on the true correct saliency map, which significantly resulted in incorrect responses, the same procedure was repeated, but here the number of incorrectly classified trials from each condition was used in the sub-sampling to generate 1000 random incorrect saliency maps. Then a similar comparison was made between these maps and the true incorrect saliency map. The latter map has to be obtained as was the true correct saliency map but with incorrect trials.

To highlight the diagnostic and anti-diagnostic regions of car 1, the relevant car 1 image (i.e. on the same condition) was overlaid on the correct saliency map to obtain a ‘car-overlaid correct saliency map’. Then, the diagnostic and anti-diagnostic regions were highlighted respectively by red and blue colors as shown in Figs 5 and 6. This procedure was repeated for each variation condition of car 1, 2 and 3, for each experiment and subject separately.

For calculating the pooled saliency maps across subjects (e.g. Figs 12 and 13), trials from different subjects were pooled and the whole above procedure was repeated on the pooled dataset.

### Computational model

To see if a hierarchical feed-forward structure could develop feature-based object recognition strategies similarly to humans, we used a previously developed brain-plausible deep convolutional neural network model of human ventral visual processing. Although not ever been provided with any biological information during training, the model has been very successful in predicting primates’ object representations at higher visual areas such as V4 and IT^2,3,43^. The model, known as ‘AlexNet’, is an eight-layered hierarchical structure (i.e. the 7^th^ layer is regarded as the model output here as the 8^th^ layer was dedicated to classification scoring on ImageNet) which extracts information from input images by applying on them several mathematical operations such as convolution, max-pooling, regularization, etc. These operations provided the infrastructure for extracting simple to complex visual features from input images along the layers. The model had been trained to be evaluated on the ImageNet Large Scale Visual Discrimination Challenge (ILSVRC) (http://www.image-net.org/), and had outperformed the state-of-the-art machines on the task. The model was developed by Krizevsky *et al*.^20^ and we used its Matlab implementation, provided by Vedaldi *et al*.^44^ (available at http://www.vlfeat.org/matconvnet/), with the same training weights learned on the ILSVRC to provide representations of the car exemplars in current study. The ILSVRC included the car categories used in the current study and around 1000 more object categories. This very large number of categories which was used in the training of the model made the model an excellent candidate to explain the generality of the brain.

A template-matching approach along with a winner-take-all algorithm was used to obtain the saliency maps for the computational model as they were calculated for humans. For that purpose, we used the representations obtained from the 3^rd^ and the 7^th^ model layers and analyzed them separately. The reason for these choices was that the 3^rd^ model layer, which was convolutional, provided the closest discrimination performance to those obtained from humans among other model layers (i.e. the discrimination rates averaged across the two datasets were respectively 65.32%, 73.24%, 77.78%, 78.08%, 79.59%, 80.25% and 81.05% correct for the first to last layers of the model on the same masked objects presented to humans). This closeness was preferred to avoid any potential influence of performance on the analyses. Moreover, the 3^rd^ layer seemed to be a suitable choice to provide insight into the intermediate-complexity feature extraction process starting at the model input (i.e. here the observer plays the role of the model’s input layer as it processes pixel-space information) and ending at the model output. The rationale behind the choice of the 7^th^ model layer was that, as explained earlier, this layer has shown to predict high-level human representational space with high accuracy^2,6,21,38^; therefore, this layer might provide highly brain-plausible processing strategies. In addition, it was a representative for the most complex visual features which could be extracted in the visual hierarchy. To perform the template matching, we applied both the masked and unmasked object images to the model and obtained their corresponding representations from the 3^rd^ and 7^th^ model layers. We fed the model only with images presented to humans in the psychophysical experiments to avoid potential differences in results. On each dataset, using Pearson linear correlation, the similarity between each of the masked and unmasked object representations was measured (i.e. conditions shown in Fig. 1B). Then, the winner-take-all algorithm decided on the category of the masked image, by comparing the 26 correlation values. Accordingly, on the HSD dataset, the masked image belonged to car 1, if the maximum correlation value was observed between its representation and any of the 13 unmasked representations of car 1. Otherwise, the masked image belonged to car 2. As the computational model processed the masked images differently from humans, the correct and incorrect trials which were obtained from the model analyses did not necessarily follow human correct/incorrect trials. In other words, the trials which were correctly recognized by humans were not necessarily recognized correctly by the model and vice versa. To reach the saliency maps of the computational model, the same Bubbles method explained earlier was repeated on the model data. The significance of the saliency maps was also evaluated as explained earlier.

### Ideal observer

Saliency maps were also calculated for an ideal observer^39,42^. The ideal observer evaluated the importance of different object parts based on the pixel space information they provided for recognition. In other words, the ideal observer, and the following classification algorithm, used original masked images to calculate the saliency maps as opposed to the computational model which used the transformed versions of the same images (i.e. object representations). Accordingly, the masked images of objects were compared with the unmasked images for their correlations in the same way as done for the computational model. The same winner-take-all algorithm was used for classification. As the ideal observer provided higher recognition performance compared to humans, to remove potential bias when comparing them, we added Gaussian noise (with standard deviations equal to 100% and 90% to the average image luminance respectively for the HSD and LSD) to the stored unmasked images to equalize the observer performance (77.45% and 75.48% respectively on the HSD and LSD) to that obtained from humans. Other implementation details of the ideal observer resembled those obtained from the computational model, including how the significance of the saliency maps was calculated for the observer.

### Data availability

The data sets generated during and/or analyzed during the current study are available from the corresponding author on reasonable request.

## Results

This study was aimed at investigating the feature-based mechanisms involved in human invariant object recognition. For that purpose, 21 human subjects participated in two object discrimination experiments in which they observed occluded images of cars. Subjects had to discriminate between a pair of cars based on their unmasked parts. The correctness of subjects’ answers revealed the relative importance of the unmasked visual features in recognition and the strategy which was used when the objects underwent different variation conditions. The only difference between the two experiments was the set of stimuli used. Designing two experiments with different levels of object similarity provided the opportunity to compare the human recognition strategies against those observed in rats which showed that object similarity could alter the invariance of diagnostic object features across variations^37^.

The similarity of objects in the two datasets (Car 1 vs. Car 2 and Car 2 vs. Car 3) was assessed in the pixel space using normalized Euclidean distance on every variation condition (Table 1). Results showed a significantly (p < 0.01, Wilcoxon signed rank test) higher similarity between the two exemplars of the first stimulus set (mean = 0.155, SD = 0.015) compared to the second stimulus set (mean = 0.187, SD = 0.008). Accordingly, we termed the first and the second image sets respectively as the ‘High similarity’ (HSD) and ‘Low similarity’ datasets (LSD) in the rest of the paper.

**Table 1:** Normalized Euclidean distance between car exemplars in the two datasets.

Variation													
Stimulus set	**Default**	**Position**			**Size**			**In-plane rotations**			**In-depth rotations**		
		**3°**	**7°**	**13°**	**6°**	**14°**	**17°**	**−40°**	**60°**	**180°**	**−40°**	**40°**	**180°**
Stimulus set 1 (Car 1 & Car 2)	0.153	0.153	0.153	0.153	0.149	0.154	0.154	0.202	0.157	0.149	0.154	0.134	0.150
Stimulus set 2 (Car 2 & Car 3)	0.194	0.194	0.194	0.194	0.189	0.195	0.194	0.176	0.174	0.187	0.134	0.186	0.179

### Human performance at discriminating masked cars

Humans were very successful at discriminating the cars using the masked images. The average discrimination rates were 76.38% (SD = 10.01) and 75.17% (SD = 2.21) across subjects respectively on the HSD and LSD, which were significantly (p < 0.001, Wilcoxon signed-rank test) above the chance level (i.e. 50%). This shows that, while humans could use the information exposed from the transparent pores to discriminate the cars, the adaptive algorithm was effective at keeping the accuracy at around 75% correct for the Bubbles analysis.

**Figure 4:**
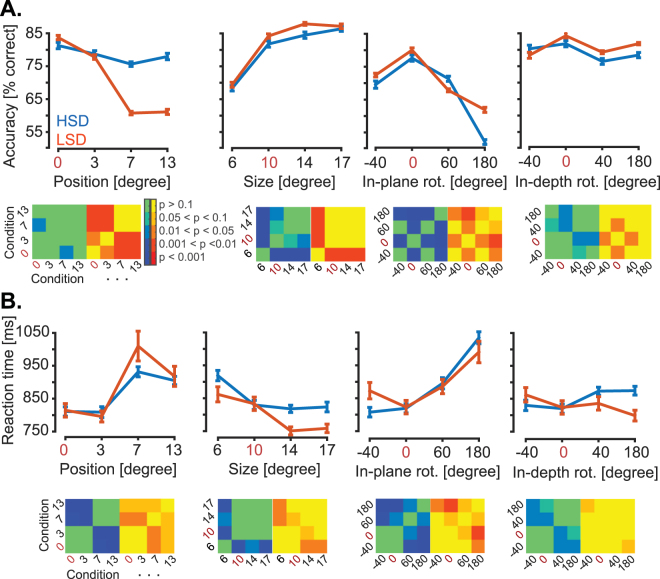
Humans’ behavioral performance on the two experiments. (**A**) Discrimination accuracy in each variation condition shown in Fig. 1. (**B**) The corresponding correct reaction times for the same variation conditions as shown in (**A**). Blue and red bars show results for the HSD and LSD, respectively. Red numbers indicate the default condition which is repeated in all graphs for better visualization. Error bars show the standard error across subjects. Accordingly, the cross-condition significance matrices with blue-green and red-yellow spectra which are provided below each graph are for the HSD and LSD, respectively. Color codes used in the matrices indicate the significance of difference between the conditions and is defined by the color bar.

The average correct reaction times (i.e. calculated over correctly answered trials) were 871 ms (SD = 172 ms) and 888 ms (SD = 217 ms) respectively on the HSD and LSD across subjects, which repeated several studies reporting the human reaction times in object categorization^4,6^. However, the discrimination accuracy was not equal in all variations and their constituent conditions (Fig. 4A). On the HSD (and LSD), the average correct rates were 78.39% (66.54%), 79.74%, (80.99%) 71.31% (71.05%) and 78.68% (80.37%) respectively for the position, size, in-plane and in-depth variations across subjects. The subjects’ accuracy was significantly (p < 0.05, Wilcoxon signed-rank test) above chance in all variation conditions. Position conditions resulted in significantly lower correct rates on the LSD compared to HSD (p < 0.001, two-tailed unpaired *t*-test). The conditions of the in-plane rotation imposed more difficulty in discrimination compared to other variations, especially when cars were oriented upside down (Fig. 4A). Figure 4 also provides cross-condition significance matrices indicating the significance of differences across condition pairs. For example, to obtain each element of these cross-condition significance matrices on the HSD, we evaluated the difference between the 13-element vectors containing accuracy values/correct reaction times of the 13 subjects in two conditions of a single variation. To obtain the level of significance between two conditions, we applied Wilcoxon signed-rank test on the two 13-element vectors. The resultant p-values were Bonferroni corrected and reported, by color codes, in the matrices provided below corresponding accuracy/reaction time plots.

Subjects’ performance has significantly (p < 0.05, two-tailed unpaired *t*-test) dropped when the cars were positioned above the central fixation point compared to when they were presented centrally on both the HSD and LSD. Subjects showed a significant performance decline in the most peripheral position condition on the LSD. Based on the accuracy and reaction times of the subjects in position conditions we can conclude that the subjects did not make saccadic eye movements towards the cars in non-default conditions (Fig. 4). Subjects showed a high performance deficit when the objects were presented in the smallest size condition (i.e. 6 degrees of visual angle) on both the HSD and LSD, with significant effects (P < 0.05, two-tailed unpaired *t*-test) on both the accuracy and reaction time. However, the result still remained significantly above the chance.

As previously reported in a more generic discrimination task with unmasked objects^45^, in-plane and in-depth rotations seem to depend on a reference-based transformation process. This process is proposed to transform the perceived object to a reference frame in which the memorized representations of objects are stored to find the best match^6^. This might be the reason behind the results of the in-plane and in-depth rotations which provided a symmetric curve around the default condition for accuracy (Fig. 4A). For the in-plane rotations, the performance dropped (i.e. accuracy decreased and reaction time increased) as the objects underwent from 0 to 180° and increased as it came closer to the default condition on both datasets. The performance was significantly (P < 0.05, two-tailed paired *t-*test) lower when the cars appeared upside down, which might be because of lack of informative visual features in that condition. Results of in-depth rotation showed a decrease in performance when the cars were mirrored (i.e. were rotated 180°) on the HSD or shown from the back view, rather than when they were presented frontally on both datasets. Humans accuracy showed a significant negative correlation with correct reaction times (r = −0.81, p < 0.001, Pearson linear correlation). This implies that variations affected both the accuracy and speed of recognition, and not one at the cost of the other^6^.

Although some conditions showed significant impacts on the discrimination performance, humans achieved correct rates which were significantly higher than those which could be achieved by chance (p < 0.001) (the significance level was p < 0.05 for the 180° in-plane rotation condition on HSD). This remarkable performance implies that humans used an invariant strategy when trying to recognize objects under variations; a strategy which was not easily affected by variations. In the following sections we investigate the feature-based mechanisms which might underlie this strategy.

### Role of diagnostic features in object discrimination

**Figure 5:**
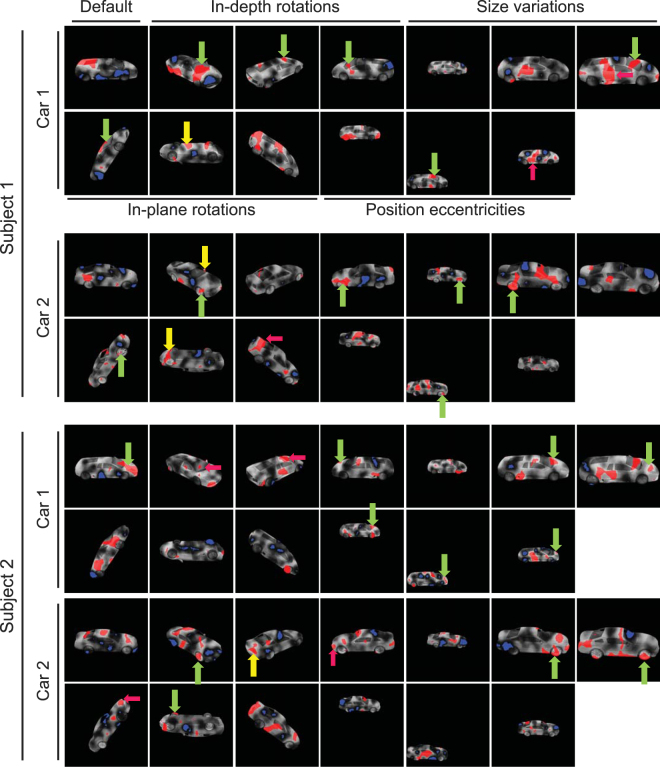
Diagnostic (red regions) and anti-diagnostic (blue regions) features found for 2 sample subjects on the HSD experiment. The brightness of car regions indicates the importance of the region in its discrimination with brighter regions leading to more accurate answers. Diagnostic features which were consistently/rarely used by subjects across variations are indicated by green/yellow arrows. Magenta arrows indicate the diagnostic features which were used by both sample subjects on the same variation condition. The 3D car models used to generate these images are available under a Creative Commons Attribution-ShareAlike 3.0 Unported License (https://creativecommons.org/licenses/by-sa/3.0/) and were freely downloaded from (https://grey.colorado.edu/CompCogNeuro/index.php/CU3D).

**Figure 6:**
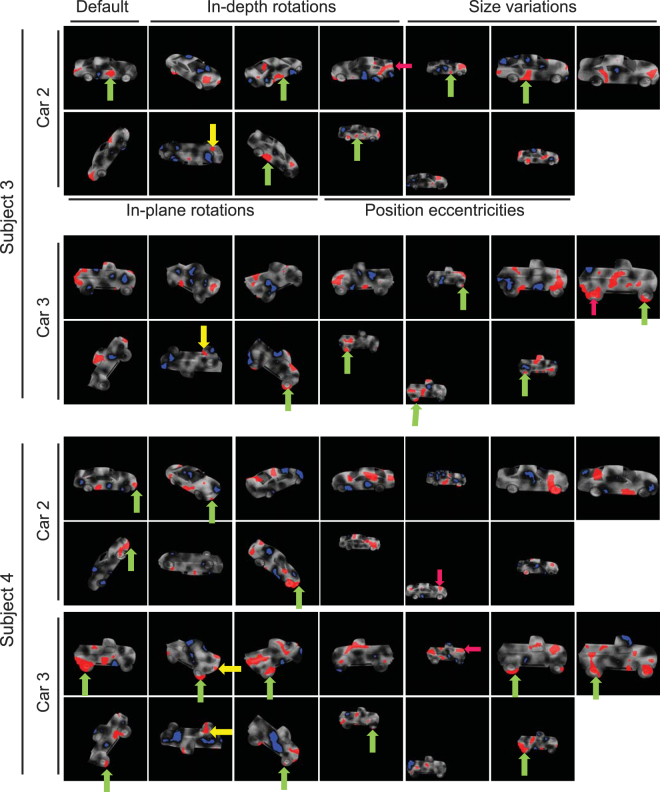
Diagnostic (red regions) and anti-diagnostic (blue regions) features found for 2 sample subjects on the LSD experiment. The details are the same as in Fig. 5. The 3D car models used to generate these images are available under a Creative Commons Attribution-ShareAlike 3.0 Unported License (https://creativecommons.org/licenses/by-sa/3.0/) and were freely downloaded from (https://grey.colorado.edu/CompCogNeuro/index.php/CU3D).

Using the subjects’ correct and incorrect responses and the Bubbles method, we extracted saliency maps for subjects in all variation conditions (Figs 5 and 6). Results for two sample subjects on the HSD are shown in Fig. 5, for different variation conditions. The brightness of the pixels on car images represents the probability of the object region to lead to a correct answer when it is visible through the bubbles mask, with brighter pixels indicating higher probability. Red and blue areas indicate regions which significantly led to correct and incorrect answers, respectively, which we termed ‘diagnostic’ and ‘anti-diagnostic’ regions as explained earlier. Note that, we increased the gray level of the windshield and side windows of Car 1 for improved visualization, whereas the actual gray level was zero in the experiments as shown in Fig. 1.

Visual inspection of the saliency maps of the selected subjects revealed several key characteristics of feature-based recognition in humans. Results of the default condition (Fig. 5, the top left-most image in each panel) suggested that not all car parts contributed equally to its correct recognition. In other words, there were areas of diagnostic features which significantly contributed to the recognition of the car whereas some other areas led to incorrect trials. For a detailed definition of car parts see Supplementary Table S1. For instance, subject 1 relied on the rear window as well as trunk and rear post of car 1 and relied on rear as well as a small portion of the front fenders of car 2 for correct recognition. A high proportion of front and rear fenders and doors of car 1 were anti-diagnostic for subject 1 while the anti-diagnostic regions of car 2 included areas on the hood, trunk, roof, front posts, doors and front bumper. For subject 2, the headlights, hood, windshield, front bumper as well as a small proportion of the doors and windows were diagnostic of car 1. Subject 2 relied on the hood, roof and rear window of car 2 for correct recognition. Proportions of the doors and front fender of car 1 and the doors, rear wheels, middle post, windshield and front bumper of car 2 were anti-diagnostic in default condition.

In the default condition of the LSD (Fig. 6, the top left-most image in each panel), the doors, rear fender and rear bumper of car 2, and the roof, hood, box and rear fender of car 3 were diagnostic for subject 3 while subject 4 used the front bumper and the doors of car 2 as well as the doors, box, rear wheels and the rear fender of car 3 as diagnostic features. These sample subjects showed a variety of anti-diagnostic regions on the default condition of the HSD.

Although there were instances of agreement on the diagnostic regions between the sample subjects (as indicated by the magenta arrows in Figs 5 and 6), results showed a high level of inter-subject variability in diagnostic and anti-diagnostic features. This will be quantitatively evaluated and compared between the two datasets in the following sections.

Next we investigated the consistency of diagnostic features across variation conditions to see whether a set of view-invariant or view-specific diagnostic features were deployed by humans for accurate recognition. On the HSD, subject 1 deployed diagnostic features from different parts of car 1 depending on the condition of the car (Fig. 5, top panel). These included the windshield, bumpers, wheels and even the car floor. Although majority of diagnostic and anti-diagnostic regions were not repeated across the variation conditions, consistent diagnostic features were also used by subjects in discrimination (as indicated by green arrows in Figs 5 and 6). These included the windshield of car 1 and the front wheels of car 2. There were also areas of diagnostic features which were only adopted when they became available to subjects (yellow arrows). These included the floor of both cars as well as the outside mirrors of car 2. For subject 2, the hood and front wheels were respectively used from cars 1 and 2 and the tail lights of car 2 were only used when they became available to the subject.

On the LSD, regions of the doors of car 2 and the wheels of car 3 were repeatedly chosen as diagnostic across variation conditions by subject 3. For subject 4, the front bumper and wheels were consistently chosen respectively from cars 2 and 3 in recognition. As for alternative features, the car floors were chosen by subject 3 on the LSD, on the upside down condition in which the consistent features were absent. The grille and the floor of car 3 became diagnostic as soon as they appeared to help subject 4 in recognition.

**Figure 7:**
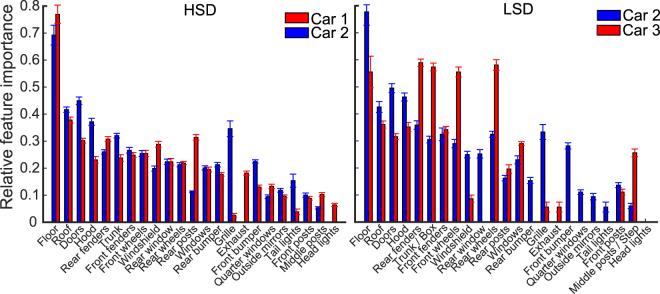
Relative importance of different car parts in recognition on the HSD (left) and the LSD (right). Relative importance is calculated as the ratio of the number of times the part included a diagnostic region to the number of times the car part had been visible in the unmasked variation conditions. Error bars show the standard error across subjects.

Consistent reliance on specific car parts across variations suggested that some car parts might have been more informative than others. For example, car wheels were consistently chosen as diagnostic features by many subjects (e.g. wheels of car 2 used by subjects 1, 3 and 4, and wheels of car 3 by subject 3) while other parts such as the windshield was considered diagnostic much fewer times by the subjects (e.g. subject 1, car 1). Therefore, next we evaluated the importance of different car parts in recognition by counting the number of times the part was diagnostic and divided the resultant number by the number of conditions in which the car part was available to the subject. This resulted in a vector of 23 numbers for each car (representing the 23 car parts shown in Supplementary Table S1) that was normalized to 1 which explained the relative importance of different car parts for each subject. This procedure was repeated for every car exemplar, dataset and subject. Figure 7 shows the across-subject averaged results of the two datasets. We sorted the car parts according to their average relative importance for the two cars on the HSD.

Results from both the HSD and LSD showed the highest relative importance for car floors which appeared only once across variation conditions when the cars were oriented upside down. Other car parts such as the roof, doors, hood, rear and front fenders provided the next highest relative importance and parts such as the headlights, middle and front posts, tail lights, outside mirrors and quarter windows showed the lowest relative importance. It should be noted that zero-valued bars in Fig. 7 show results of either the car not possessing the part (e.g. rear bumper for car 3) or the part not ever including diagnostic regions (e.g. headlights for car 2). The two cars in HSD largely agreed on the level of relative importance of their parts. However, this is not true in the case of several parts such as the rear posts, grille and headlights. On the LSD, the number of parts which showed different relative importance between cars 2 and 3 increased since the cars were structurally much more different than those in the HSD (Table 1). Examples include the rear fender, front and rear wheels and the trunk/box of car 2/3 which provided a significantly higher relative importance for car 3 than for car 2. Qualitatively, a similar order of relative importance was observed for car 2 parts on both HSD and LSD. The across-feature grand average relative importance for car 2 in the HSD and LSD were respectively 0.24 (SD = 0.22) and 0.27 (SD = 0.23) which were not significantly different (p = 0.19, unpaired two-tailed *t*-test). It seems that, one key parameter which determined the relative importance of the car parts was the relative size of the parts (e.g. compare the area of the roof with that of the middle posts). However, the relative size of the car part per se failed to explain many instances of relative importance of car parts shown in Fig. 7. For instance, compare the relative importance of the exhaust with the quarter window of car 1, the grille with the windows of car 2 and the front wheels with the doors of car 3. Therefore, it seems that humans deployed other recognition mechanisms which gave systematic weights to informative visual features for accurate recognition. A more quantitative evaluation on the consistency of the visual features will be provided in the following sections.

In conclusion, these results revealed that humans relied on a set of diagnostic features to recognize the cars. A portion of these features were consistently chosen by subjects across several variation conditions (the features indicated with green arrows in Figs 5 and 6), while some of them were used only rarely (the features indicated with yellow arrows in Figs 5 and 6) as soon as they appeared. Results also showed a high level of inter-subject variability in the choice of diagnostic features, meaning that individuals developed their own personalized strategy in object recognition.

### Impact of object similarity on human object discrimination

In order to investigate whether humans’ reliance on diagnostic features could be explained by a view-invariant or view-specific strategy, we measured the amount of overlap between the diagnostic regions found for different variation conditions using the method developed by Alemi-Neissi *et al*.^36^. To perform this analysis, we considered only conditions from the affine variations of size, position and the default condition and computed the amount of overlap between every possible pair of these conditions (i.e. 11 pairs of conditions were accounted for each car in which the diagnostic features obtained from the conditions could possibly overlap). We calculated the overlap in two circumstances: ‘raw overlap and aligned overlap’. ‘Raw overlap’ refers to calculating the pixel-space overlap between diagnostic regions obtained from the same car appearing in a pair of different views (variation conditions), while ‘aligned overlap’ refers to the circumstance of overlap calculation in which the two different views (variation conditions) of the same car are reverse-transformed to the default condition prior to overlap calculation. As an example, consider the case of a pair of conditions in which the cars (i.e. the same cars) were respectively in 3° position and 14° size conditions. In the case of raw overlap, the pixel-space overlap was calculated without any manipulation of car images using equation (1) knowing only the regions of diagnostic features of both conditions. In the case of aligned overlap, however, the first car was returned to the center of the screen and the second car was down-scaled to the default size, so that reverse-transformed images of the cars perfectly overlapped in the middle of the screen. In the latter case the diagnostic features of the car views might or might not overlap depending on the feature-based recognition strategies adopted by humans and the computational models. Finally he amount of overlap between the car view in both the raw and aligned circumstances was calculated according to equation (1):

1 $$Op=\frac{Oa}{Oa+Da1+Da2}$$

where ${Oa}$ refers to the diagnostic car areas (i.e. number of pixels) which overlapped between the first and the second variation conditions, ${Da}1$ and ${Da}2$ refer to the area of diagnostic regions for the variation conditions 1 and 2, respectively, and ${Op}$ is the proportion of the overlapping area of the superimposed conditions. It should be noted that, we used affine transformations since they could be correctly reverse-transformed in the aligned overlap case and lead to a single object as a result. As it was shown in Fig. 1B, other transformations were not affine (i.e. cars had several parts which were visible in one condition but invisible in others). Therefore, even if reverse-transformed, the diagnostic features could not possibly overlap.

**Figure 8:**
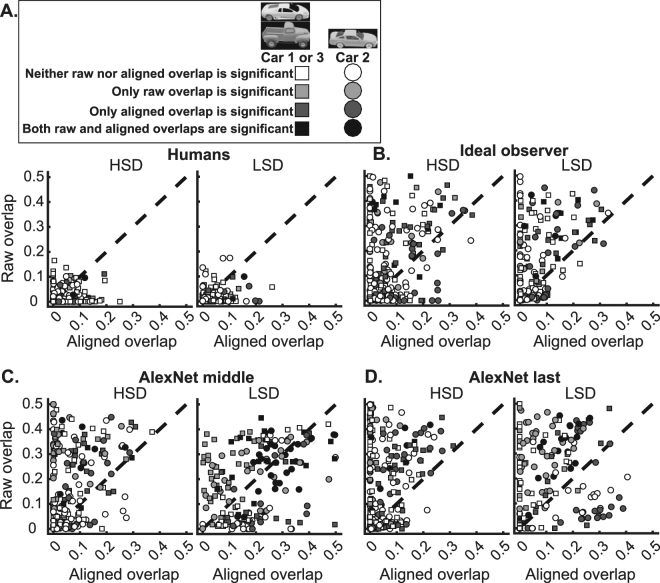
Scatters of raw versus aligned overlaps between diagnostic regions. Results from humans (**A**), ideal observer (**B**) middle and (**C**) last layers of the computational model (**D**), on the HSD (left) and LSD (right) experiments are shown. The overlaps were measured using the procedure explained in section 3.3. Raw overlaps are plotted against aligned overlaps for each of the 21 pairs of conditions (pool of zero and non-zero overlapping conditions) of the two cars. These results are from size and position variations, on which the reverse-transformation could result in totally overlapping diagnostic regions in ideal cases. The 3D car models used to generate these images are available under a Creative Commons Attribution-ShareAlike 3.0 Unported License (https://creativecommons.org/licenses/by-sa/3.0/) and were freely downloaded from (https://grey.colorado.edu/CompCogNeuro/index.php/CU3D).

In order to determine the significance of the measured overlaps we used the method proposed by Nielsen *et al*.^17^. Read that paper for details of the method. Briefly, in this method we first calculated the minimum box which encompassed each object view. Then, in both raw and aligned cases, the set of diagnostic regions found for each object (stuck together) was moved to random positions within its surrounding box and the area of overlap was calculated between the first and second object views using equation (1). Note that, in the case of aligned overlap, the encompassing boxes of the two object views totally overlapped as the objects did after reverse-transformation. The random positioning of the diagnostic features was repeated 1000 times and the random overlaps were recalculated. Then, a one-tailed permutation test determined the significance of the true overlaps against the resultant null distribution. The overlaps were considered significant if their values were higher than the 95^th^ percentile of the overlaps calculated from the random positioning (P < 0.05, Fig. 8A). The data points (which were obtained by plotting raw and aligned overlap values on the same condition pairs against each other) for car 1/3 and car 2 are indicated with squares and circles in Fig. 8, respectively. Shades of gray indicate the level of significance of raw and aligned overlaps against the 1000 random overlaps. White and black shades represent cases in which none and both overlaps were respectively significant while gray and dark shades indicate cases with only raw and aligned overlap being respectively significant.

203 (out of 286) pairs of conditions showed non-zero overlapping areas of diagnostic features across the entire set of subjects (i.e. 13 subjects) on the HSD. As shown in Fig. 8A, left, on the HSD, the vast majority of overlapping pair of conditions (143 out of 203, 70.4%) fell onto the area beneath the dashed line meaning that they had higher aligned overlaps (mean = 0.028, SD = 0.042) than raw overlaps (mean = 0.012, SD = 0.025), the difference of which was significant when compared (p < 0.01, Wilcoxon signed-rank test). Many pairs of conditions (81 out of 203, 40%) showed non-zero values of aligned overlap with zero raw overlaps. A number of cases (31 out of 203, 15.3%, dark data points) showed significant values of aligned overlaps but insignificant values of raw overlaps. Only 6 cases (out of 203, 3%) showed significant values of raw and insignificant values of aligned overlaps. In only 2 cases (out of 203, 1%) both the raw and aligned overlaps were significant.

On the LSD, as Fig. 8A, right, shows, most of the data points (115 out of 164, 70.1%) were located below the dashed line showing higher aligned overlaps (mean = 0.031, SD = 0.044) than raw overlaps (mean = 0.017, SD = 0.027), whose difference was also significant when compared (p < 0.05, Wilcoxon signed-rank test). In many cases (59 out of 164, 36%), the data points showed non-zero values of aligned overlaps while their corresponding raw overlaps were zero. In a large proportion of cases (36 out of 164, 22%, dark data points) significant overlaps for the aligned features were observed along with insignificant overlaps for the raw features. Only six cases (out of 164, 3.65%) showed significant overlaps in the raw and insignificant overlaps in the aligned conditions. In only two cases (out of 164, 1.2%) both the raw and aligned overlaps showed significant values.

**Figure 9:**
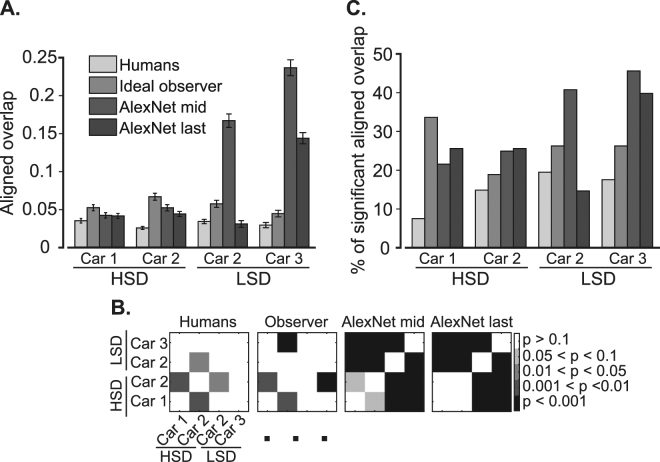
Quantitative comparison of diagnostic features’ consistency between humans and computational models. (**A**) Across-subject averaged aligned overlap from different modalities (including humans, observer, and model), and (**B**) the corresponding cross-object and cross-experiment significance matrices which indicate the level of significance in differences (as evaluated by unpaired two-tailed *t*-test) in color codes. (**C**) Percentages of the number of condition pairs which provided significant overlap values. Error bars show the standard error across subjects.

These results show a significant advantage for aligned overlaps compared to raw overlaps, which adds support to a view-invariant strategy (in which subjects relied on a set of diagnostic object features and used them despite changes in object viewing conditions) rather than a screen-centered strategy (in which specific regions of the screen relative to the object were relied on for recognition which could lose their importance if the object condition varied on the screen) used by humans in object recognition. The averaged aligned overlaps across subjects as well as the percentages of aligned overlaps on the HSD and LSD are summarized in Fig. 9.

Average aligned overlaps across subjects (light gray bars in Fig. 9A) on the HSD were 0.035 and 0.025 respectively for cars 1 and 2, and 0.034 and 0.029 respectively for cars 2 and 3 on the LSD. Although the pooled results from the HSD did not show a significant difference (p = 0.63, unpaired two-tailed *t*-test) with those from the LSD, the average aligned overlaps of car 2 on the LSD showed a significantly higher value compared to its value on the HSD (Bonferroni corrected p = 0.013, two-tailed *t*-test, Fig. 9B the left-most cross-object significance matrix). This implies that the lower similarity between the objects, which were discriminated, increased the consistency of the diagnostic features across variation conditions. The evaluation of the percentages of aligned overlaps (Fig. 9C, light gray bars) also showed an increase from 11.55% on the HSD to 19.2% on the LSD (averaged across cars in the same image set). These were especially interesting for car 2 which were 15.4% and 20.2% respectively on the HSD and LSD; showing 31% improvement. Together results of Fig. 9 show a shift of strategy from a more variation-dependent to variation-independent strategy in the choice of diagnostic features when discriminating two dissimilar (LSD) cars compared to relatively similar cars (HSD). This is reflected in both the amount of aligned overlap as well as the percentages of significant overlaps of the diagnostic features from car 2 which participated in both experiments.

**Figure 10:**
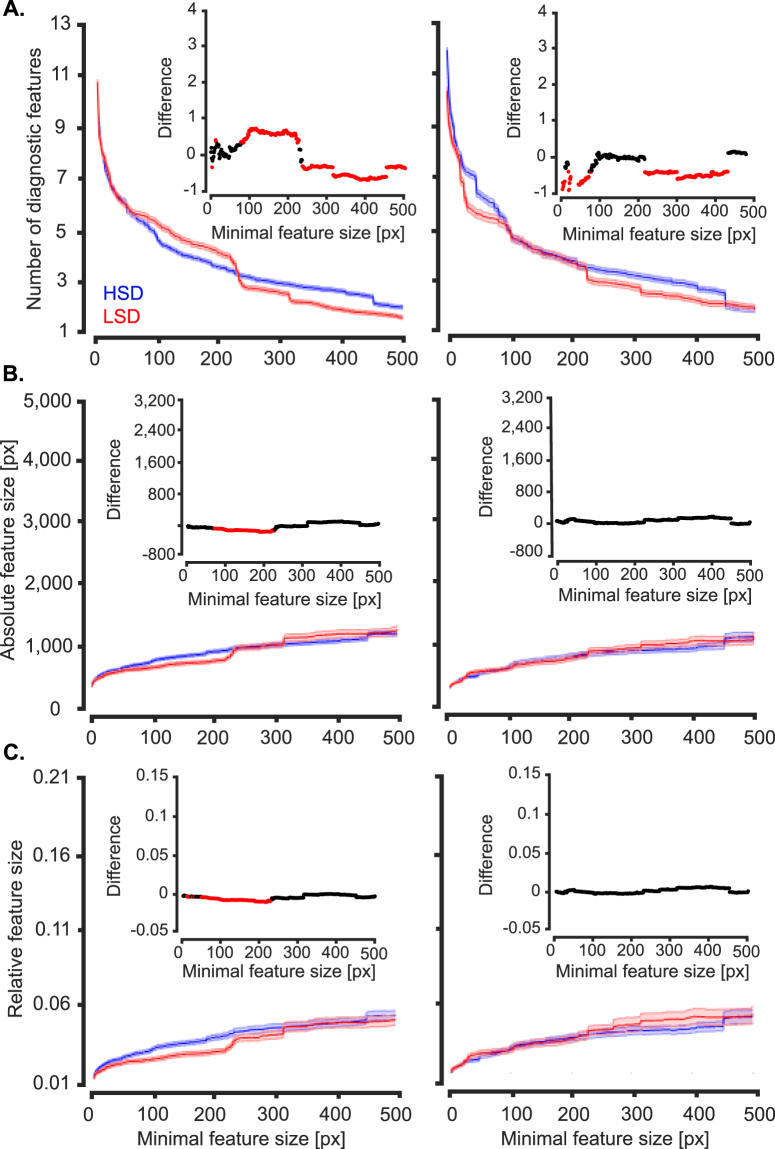
Average number and size of diagnostic features found on the two experiments. (**A**) Average number of diagnostic features obtained from all variation conditions of the two image sets (i.e. HSD and LSD) as a function of the minimal feature size. Minimal feature size was defined as the size threshold below which features were considered in the analysis. Results on the left column were computed by pooling across all objects, variation conditions, features and subjects participating in each experiment. Shaded regions indicate the standard error across all the mentioned dimensions. The right column shows the same results but only for car 2. (**B**) Average feature size and (**C**) relative feature size (as obtained by dividing the size of each feature by the area of the corresponding car view). Insets show the deference between the values obtained from ☺ the two image sets (with black showing no significant difference and red showing significant difference at p < 0.05, two-tailed *t-*test).

To gain a deeper insight into other potential differences between the recognition strategies of humans when discriminating object exemplars with different levels of similarity, we evaluated the numbers and sizes of diagnostic features obtained from the two experiments (i.e. HSD and LSD). As it was obvious in the results (Figs 5 to 7), the diagnostic features varied in the size of area that they covered, ranging from several to hundreds of pixels. Therefore, as previously suggested^37^, we evaluated the numbers, sizes and relative sizes of diagnostic features as a function of minimal feature size to see if a systematic difference existed between the two experiments. The minimal feature size provided a threshold which determined the size of the smallest diagnostic feature which could be included in the analyses. Therefore, values of the horizontal axes in the plots of Fig. 10 indicate the minimum size of diagnostic features considered in the analysis. To produce the results shown in Fig. 10, we pooled the parameters (i.e. the number, size and the relative sizes of the features) obtained from all variation conditions and subjects and calculated the average results in the corresponding experiment. The significance of differences between the statistics (size, number and relative size) of diagnostic features from the HSD and LSD were compared using two-tailed unpaired *t*-test and their p-value results are provided in the insets plots in Fig. 10.

As the results show (Fig. 10A), a significantly (p < 0.05) larger number of diagnostic features were found which had medium sizes between 78 to 227 pixels on the LSD compared to HSD. However, the large-size diagnostic features of the HSD, with areas larger than 236 pixels, significantly outnumbered the features with the same area found on the LSD. The advantage of the HSD, which asymptotically became insignificant for sizes larger than 1357 pixels, along with the advantage for the LSD in small to medium sizes suggest that the average number of diagnostic features for the two datasets were roughly equal. However, the crossover of the average curves at around the minimal feature size of 250 pixels revealed a larger number of features with smaller areas for the LSD and larger areas for the HSD, respectively. Interestingly, car 2, which participated in both experiments, showed a significantly lower number of features at both small (from 1 to 78 pixels) and large (from 228 to 451 pixels) scales on the HSD compared to LSD. This result revealed a decrease in size of the diagnostic features as a result of lower similarity between categories in the discrimination task, which is consistent with previous reports from rats^37^.

Next we evaluated the absolute and relative sizes of the features found on the two experiments (Fig. 10B and C). To calculate the relative sizes of the features, the absolute size of a given feature was divided by the area of the car in the corresponding variation condition. Although averagely smaller absolute and relative sizes were observed for small features on the LSD (significant differences were observed below minimal features sizes of 225 and 232 pixels respectively for the absolute and relative feature sizes), results of car 2 showed no significant difference in feature sizes between the HSD and LSD. In conclusion, this implies that the absolute and relative sizes of the diagnostic features were not significantly different between the HSD and LSD for car 2 which participated in both experiments, and the observed significant differences observed for the exemplar-averaged results (Fig. 10B and C, left) were mainly caused by the properties of the other exemplars in the datasets (i.e. car 1 and 3 respectively on the HSD and LSD) rather than differences in the recognition strategies between the datasets. Therefore, the similarity of objects in the dataset did not impose a significant impact on the sizes of the diagnostic features. This is in contrast with the rats’ data which showed an increase in the feature size when discriminating less similar objects^37^.

**Figure 11:**
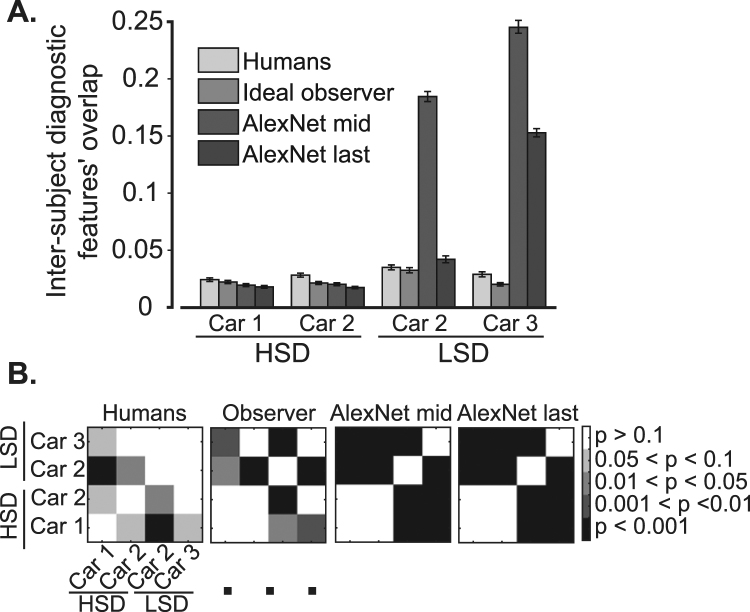
Inter-subject overlap (consistency) of the diagnostic features. (**A**) Inter-subject overlap of the diagnostic features was calculated in similar variation conditions between every possible pair of subjects participating in the HSD and LSD experiments. For the observer and the model, the inter-subject overlaps refer to calculating the overlap of the diagnostic features found on the same set of stimuli used in human experiments. The error bars indicate the standard error across subject pairs. (**B**) The corresponding cross-object cross-experiment significance matrices show the Bonferroni corrected level of differences between the overlaps calculated for different objects.

We also investigated the potential influence of exemplars’ similarity on the consistency of diagnostic features across subjects. For that purpose, we calculated the overlap between diagnostic features of the same variation conditions in all possible pairs of subjects (Fig. 11). The overlaps were calculated using equation (1). The average of overlap was significantly (p < 10^−6^, two-tailed *t-*test) higher for the LSD (mean = 0.029) compared to the HSD (mean = 0.017). Interestingly, results from car 2 showed a significant (p = 0.03) advantage for the LSD (mean = 0.035) compared to HSD (mean = 0.021), which revealed the dependence of the inter-subject consistency on the similarity of car exemplars. These results suggest that when the cars were closely matched (HSD), different subjects relied on their own sets of features which were not necessarily adopted by other subjects in object recognition, while in more dissimilar object pairs (LSD) a higher level of agreement existed between subjects when deploying informative features. The dependency of the inter-subject consistency of diagnostic features on the similarity of objects repeated previous results from rats^37^.

### Quantitative comparison of the results between humans and computational models

We provided the results of the same analyses performed on humans with those obtained from the outputs of an ideal observer and the middle and last layers of the computational model (Figs 8, 9 and 11). It should be noted that, as a subset of images were presented to each human subject, the same set of images which were presented to humans, were also presented to the ideal observer and the computational model to avoid possible bias from comparisons. In this section, we explain the results from the computational models and those obtained from humans. As explained earlier, the ideal observer implemented a pixel-space solution to the object discrimination problem while the middle and last model layers, using feature extraction mechanisms, deployed more complex features from the input images to solve the same problem. Possible correlations between human results and those obtained from the computational models can provide evidence that humans might be developing the same strategies as implemented by the models in solving feature-based invariant object recognition. Implementation details for the ideal observer and the computational model were provided in the Methods section.

### Consistency of diagnostic features across variations

We plotted aligned vs. raw overlap scatters for the ideal observer as well as the middle and last model layers on both the HSD and LSD (Fig. 8B to D). When comparing the scatter plots obtained from humans with those obtained from the observer and the model, significantly (p < 0.01, two-tailed unpaired *t*-test) higher values of raw and aligned overlaps can be seen for the observer (with means = 0.087 and 0.081), the middle (with means = 0.078 and 0.197) and last model layers (with means = 0.081 and 0.118) respectively on the HSD and LSD. However, as opposed to humans, both the ideal observer and the computational model showed higher values of raw versus aligned overlaps (i.e. much more data points are positioned above the dashed line in Fig. 8B to D, see also % Higher raw/aligned overlap columns in Table 2) on both HSD and LSD. This suggests that, rather than a view-invariant strategy in choosing diagnostic features which remained stable across variations, a low-level screen-centered strategy seems to be at work for the observer and the model compared to humans.

**Table 2:** Statistics obtained from the raw vs. aligned overlap results in Fig. 8, for observer and computational model on the two datasets.

Model	Dataset	Object	% Higher raw overlap	% Higher aligned overlap	% Dark	% Gray	% Black
Ideal observer	HSD	Car 1	63.4	36.6	9.9	6.1	0.8
		Car 2	62.4	37.6	14.9	2.6	4.2
	LSD	Car 2	70.8	29.2	12.7	4.6	2.9
		Car 3	63.5	36.5	11.5	7.5	4.0
AlexNet middle	HSD	Car 1	75.2	24.8	10.7	7.7	3.4
		Car 2	65.9	34.1	10.0	7.7	2.3
	LSD	Car 2	81.8	18.2	10.6	15.2	13.1
		Car 3	61.9	38.1	8.1	16.2	13.1
AlexNet last	HSD	Car 1	75.2	24.8	12.0	3.9	2.7
		Car 2	65.9	34.1	12.8	7.4	1.9
	LSD	Car 2	81.8	18.2	13.7	19.0	9.8
		Car 3	61.9	38.1	8.6	6.9	0

When comparing the number of the dark data points (i.e. which represent cases with significant aligned, insignificant raw overlaps), a higher percentage is observed for humans on the HSD (15.3%) and LSD (22%) compared to the observer and the computational model (Table 2). Neither the observer nor the computational model revealed an increase in the percentages of dark points as a result of change in datasets. Rather, an opposite effect was observed with a higher percentage of gray points for the observer and the model compared to humans. Searching for the effect of increased aligned overlap and increased percentages of significant aligned overlap for car 2 which was observed for humans on the LSD compared to HSD (Fig. 9, light gray bars), we observed the same increases only for the middle model layer (Fig. 9, dark bars). The inter-subject consistency also increased for middle model layer (Fig. 11, dark bars). Hence, the computational algorithms failed to repeat the feature-based strategies adopted by humans when discriminating objects with different levels of similarity.

**Figure 12:**
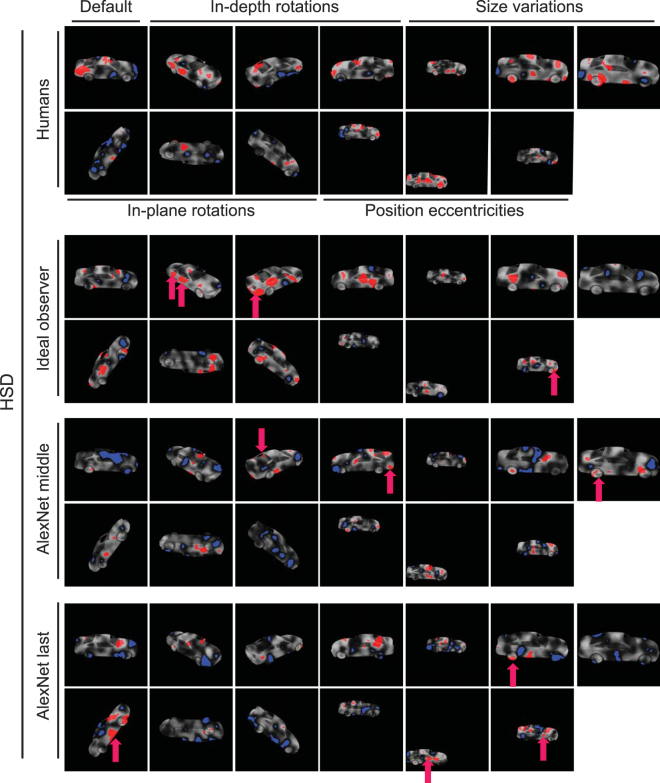
Pooled saliency maps for humans as well as computational models on the HSD. Diagnostic (red) and anti-diagnostic (blue) regions for car 2 on the HSD obtained from humans (top row), ideal observer (second panel from top) and middle/last model layers (the two bottom panels). Maps were generated by pooling the trials from all subjects. The 3D car models used to generate these images are available under a Creative Commons Attribution-ShareAlike 3.0 Unported License (https://creativecommons.org/licenses/by-sa/3.0/) and were freely downloaded from (https://grey.colorado.edu/CompCogNeuro/index.php/CU3D).

**Figure 13:**
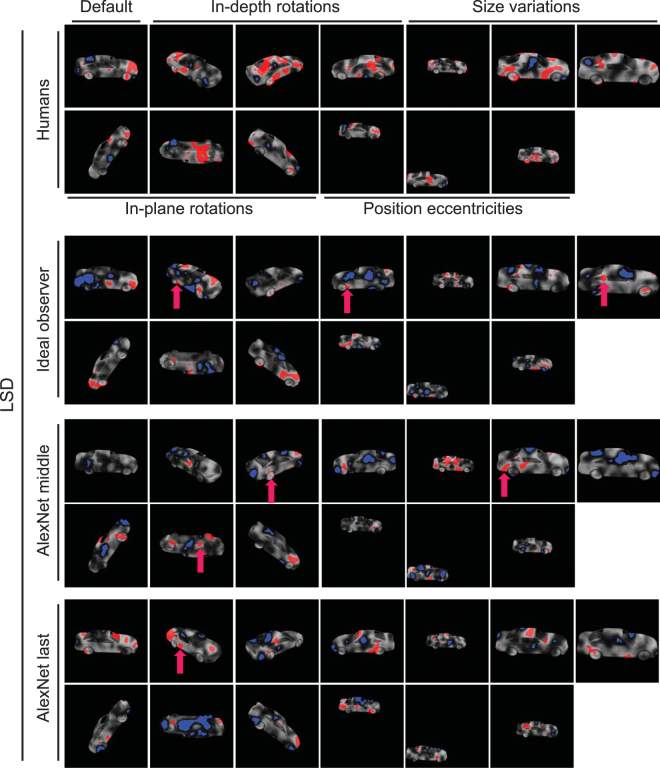
Pooled saliency maps for humans as well as computational models on the LSD. The details are the same as in Fig. 12. The 3D car models used to generate these images are available under a Creative Commons Attribution-ShareAlike 3.0 Unported License (https://creativecommons.org/licenses/by-sa/3.0/) and were freely downloaded from (https://grey.colorado.edu/CompCogNeuro/index.php/CU3D).

We also computed pooled saliency maps for humans, ideal observer and computational model (i.e. based on their responses to the same set of masked images used in human experiments). To obtain these maps, we pooled the entire set of masked images from all subjects in each experiment and repeated the same procedure as was done for individuals with the pooled correct and incorrect trials from the humans (Figs 12 and 13). Careful comparison between the pooled saliency maps obtained from the observer and model showed very limited correlations with humans. We highlighted the diagnostic features which were shared by humans and observer/computational model by magenta arrows on the relevant variation conditions in Figs 12 and 13. There were only 4, 3 and 4 diagnostic features which were shared by humans and respectively the observer, middle and last model layers, on the HSD. These numbers were respectively 3, 1 and 1 on the LSD. These qualitative results suggest that a different set of mechanisms might be developed for object recognition in humans which are not implemented by either the ideal observer or the computational model. However, a more quantitative approach was needed for more decisive conclusions.

In order to quantitatively compare the saliency maps in Figs 12 and 13, we calculated also the correlations between the pooled maps obtained from the observer and the model with those obtained from the humans (Table 3). To see whether the correlations were significant or not, we used the procedure explained earlier in the ‘Bubbles method’ to generate random correct saliency maps for the pooled human data, observer as well as model layers. Using the random sub-sampling procedure explained earlier, we calculated 1000 random correct saliency maps for the model as well as the observer whose correlations were computed and compared against the 1000 random saliency maps obtained from the human data. Finally, the true correlation values were evaluated for significance against the 1000 random correlations obtained from the random saliency maps. The true correlation value was considered significant if it was larger than 950 random correlation values (p < 0.05).

**Table 3:** Comparison between the saliency maps obtained from humans and the ideal observer, as well as the middle and last model layers.

Model			Variation												
	**Dataset**	**Object**	**Default**	**Position**			**Size**			**In-plane rotations**			**In-depth rotations**		
				**3°**	**7°**	**13°**	**6°**	**14°**	**17°**	**−40°**	**60°**	**180°**	**−40°**	**40°**	**180°**
Ideal observer	HSD	Car 1	0.10	0.06	0.07	0.12	−0.31	0.13	−0.05	−0.21	−0.26	−0.17	0.05	−0.01	−0.16
		Car 2	0.26*	−0.06	−0.20	0.11	−0.22	0.25*	−0.06	0.16	0.10	0.04	0.08	0.10	−0.10
	LSD	Car 2	−0.18	0.06	−0.05	0.16	−0.01	0.20	0.18	0.27*	−0.06	−0.02	−0.02	0.02	0.03
		Car 3	0.10	0.17	−0.24	0.14	0.05	0.10	−0.18	0.02	0.02	−0.23	−0.03	0.14	0.05
AlexNet middle	HSD	Car 1	−0.23	0.10	0.15	−0.04	−0.07	0.02	−0.13	−0.16	−0.02	0.39*	0.01	0.19	0.01
		Car 2	0.12	−0.15	0.08	0.30	−0.25	0.27	−0.03	−0.14	−0.31	−0.22	−0.10	0.16	0.16
	LSD	Car 2	−0.01	−0.03	−0.05	0.02	0.03	0.06	0.08	−0.09	−0.01	−0.08	0.03	0.09	0.01
		Car 3	0.21	0.21	0.02	0.22	0.36*	−0.02	−0.42	0.14	−0.16	−0.18	−0.16	−0.12	0.32*
AlexNet last	HSD	Car 1	0.10	−0.12	−0.07	0.25	−0.07	−0.02	−0.05	−0.09	0.25*	0.14	0.02	0.16	−0.10
		Car 2	0.39*	−0.05	−0.25	0.08	−0.05	0.06	−0.27	−0.06	−0.02	0.05	−0.18	0.17	0.12
	LSD	Car 2	0.08	0.07	0.36*	−0.18	0.10	−0.33	0.14	−0.12	−0.10	0.02	−0.16	0.03	−0.03
		Car 3	−0.07	−0.07	0.09	0.03	0.24	0.01	−0.25	−0.01	−0.05	−0.17	−0.04	−0.15	0.24

Values are correlation coefficients between saliency maps and stars indicate significant correlations.

As the correlation results show (Table 3), neither the ideal observer nor middle/last model layers showed many instances of significant correlation with the human results. A total of 9 variation conditions (out of the 156 variation conditions in Tables 3, 5.7%) showed significant correlations with humans without notable differences between HSD and LSD. Three instances were significant for the ideal observer as well as for the middle/last model layers. This little correlation in the results is of no surprise as none of the models could emulate humans’ consistency in their choice of visual features across variation conditions (Figs 8, 9 and 11).

**Figure 14:**
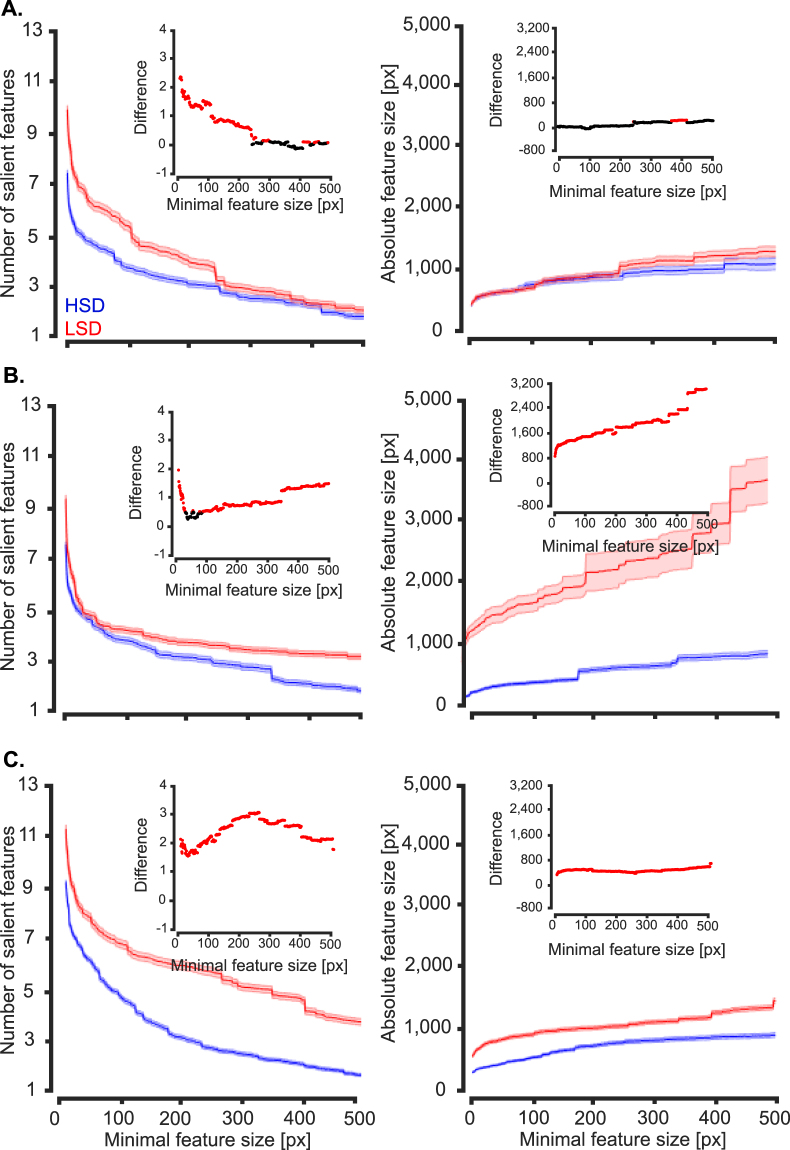
Average number and sizes of diagnostic features of car 2 in the two experiments. (**A**), (**B**) and (**C**) show the results from ideal observer, the middle and last layers of the AlexNet model, respectively. Left column: average number of diagnostic features obtained from all variation conditions of the two image sets (HSD and LSD) as a function of minimal feature size. Right column: average feature size. Shaded regions indicate the standard error. Insets show the significance of difference between the values obtained from the two image sets (with black showing no significant difference and red showing significant difference at p < 0.05, two-tailed unpaired *t-*test).

Finally, we investigated the effect of objects’ similarity on the ideal observer as well as middle and last model layers to see whether they would select a lower number of features on the LSD as was seen for humans (Fig. 10A). For the sake of brevity, we have only provided results of the number and absolute sizes of features from car 2 which included in both datasets (Fig. 14). It should be mentioned that, the results of the other objects and relative sizes of the features showed similar patterns.

As opposed to humans (Fig. 10A, right), the ideal observer, middle and last model layers showed an increase in the number of features on the LSD compared to HSD (Fig. 14, left column). However, the difference between the HSD and LSD descended as sizes of the minimal features increased for the observer, while the opposite was true for the model layers. Absolute sizes of the features were also higher on the LSD compared to HSD with increasing difference as a function of minimal feature size for the three cases (Fig. 14, right column). These results show that, both the ideal observer and computational model used a higher number of diagnostic features with larger areas. Therefore, it can be concluded that, neither a pixel-level (i.e. idea observer), nor an intermediate-complexity (i.e. middle model layer) or high-complexity (i.e. last model layer) feature extractor algorithm could emulate the human strategies when solving the feature-based object discrimination tasks of the current study.

## Discussion

Invariant object recognition, which refers to accurate and rapid recognition of objects under variations, is a distinguishing ability of human vision which has not yet found any artificial counterpart in the field of machine vision. The reason behind this superiority is the complexity of human visual processing, especially when encountering complex recognition problems^46–49^. This study investigated feature-based object recognition under common object variations in humans and two computational models of human vision. The models extracted low-, intermediate- and high-complexity visual features from object images and enabled us to search for a computational account of human visual processing at different levels of complexity.

Results showed that, rather than utilizing all car parts with equal probability, humans relied on specific parts for recognition (Figs 5 and 6). Accordingly, several parts, despite their comparable sizes to other parts, contributed more dominantly to correct recognition of the cars (Fig. 7). These results are supported by previous findings which observed intermediate-complexity feature-based strategies for object^28–30,33^ and face^50^ recognition in primates. Relying on intermediate-complexity features seems to be a logical strategy as such features have been shown to provide the richest space of information in object recognition compared to features with higher or lower levels of complexity and size^34^. While many previous studies have used Bubbles method to investigate feature-based mechanisms of vision, they have not asked how variations would change the diagnostic features of objects^28–30,33^. Therefore, current study has extended previous investigations by generating a set of variation-controlled stimuli to study whether humans use a set of view-specific or view-invariant diagnostic features across variations. Detailed computational analyses used in current study has provided evidence that, at least on the current variation-enriched image set, humans deployed a set of view-invariant diagnostic features in recognition (Figs 5 and 6). In other words, proportions of the features used in one variation condition significantly overlapped with those used across other conditions (Figs 8 and 9).

By default, the relative consistency of diagnostic features across variation conditions was less probable, since this locking of visual processing on specific object parts seems very energy-consuming compared to relying on some low-level variation-unaffected strategy across variation conditions. In fact, low-level strategies, such as reliance on screen-centered differences in object appearance, could have provided a trivial solution to object discrimination if we had failed to consider variations which drastically changed the appearance of objects on the screen. Therefore, in order to reach recognition rates which were significantly above chance (Fig. 4), subjects had to have developed strategies which were unaffected by object variations This suggests that, previous studies which did not change the object appearance on the screen^28–30^ might have suffered greatly from allowing the subjects to develop screen-centered strategies when discriminating between the objects under question.

It has been previously suggested that the similarity between objects plays a dominant role in their high-level brain representations^51–53^, and can also modulate the strategies of object discrimination^37^. Therefore, in the current study, we also investigated human object discrimination strategies at two levels of object similarity. Results showed that, as in rats^37^, humans smoothly shifted their feature-based strategy from being view-specific on the high-similarity dataset (HSD) to a rather view-invariant strategy on the low-similarity dataset (LSD). More specifically, humans used a set of diagnostic features which were more consistent and less numerous across variation conditions on the LSD (Figs 9 and 10). These similarity-dependent results imply that the level of invariance reported in many human psychophysical studies^4,6,48,49^, which has always been a point of conflict^11,54^, could have been influenced by the similarity of objects used in the discrimination task.

Our results also showed that none of the computational models used in this study could predict human feature-based object discrimination strategies and how those strategies were modulated by the level of object similarity (Figs 8, 9, 12, 13 and 14). This is interesting as recent studies have shown a high level of brain-plausibility for the hierarchical model (AlexNet^20^) used in the current paper^2,4,6,21,43,55^. It is noteworthy that the mentioned studies have evaluated the brain-plausibility of the model at the level of high-level representations and suggested that these models might use the same mechanisms adopted by the human brain in object processing^3^. Clearly, it is different from evoking the neural-level feature extraction mechanisms themselves, as was done in the current study. Many previous studies have reported discrepancy between object recognition in humans and computational models of vision^31,56,57^. Among them, a recent systematic study showed that none of the state-of-the-art computational object recognition algorithms (including AlexNet) revealed the human-like object-part sensitivity in recognition^28^. We believe that this discrepancy can be explained by the top-down cognitive processes involved in visual processing of the human brain which are absent from feed-forward computational models of vision^35,58,59^. More specifically, the dynamical strategies developed by both humans and rats might have been evoked by top-down mechanisms developed in higher visual brain areas such as PFC. Pre-frontal cortex, which plays an important role in object recognition^14,16,58^, has shown very flexible structure in representational, decisional and learning operations when encountering different object recognition tasks^60^. These PFC-related operations, if incorporated in computational models of vision, might help in finding closer matches for the human vision. As our image set was generated using occluding masks, it may have involved recurrent mechanisms of the brain in humans which are also absent from the models used here. Therefore, it seems relevant to compare the human results with those obtained from recently-developed brain-plausible recurrent models of vision^5,61^ in future studies.

One critical observation of current study is the variability of the diagnostic features across different human subjects (Figs 5, 6 and 11). This implies that every individual has relied on a set of features which they considered diagnostic, but were not necessarily considered important by other subjects. This result is in contrast with previous results from rats, which showed a higher level of inter-subject consistency of diagnostic visual features^36,37^. This discrepancy can be explained in light of the much higher computational processing power of the human brain compared to rats. Moreover, subjects of our study came from different visual backgrounds and are incomparable with the rats of previous studies^36,37^ which were bred in similar constrained environments and have been solely trained to discriminate specific visual stimuli. In addition, the car models used in the current study provide more structural differences to each other compared to the artificial objects shown to those rats^36,37^, thus providing more options of diagnostic features for human individuals to choose from. Therefore, it should not come as a surprise to observe much more individual variability of strategies among humans compared to rats. Besides, while the recognition strategies developed by rats could be accurately predicted by a pixel-level ideal observer such as the one used in this study, the recognition strategies of our subjects could not be predicted by any of those computational algorithms. These add support to the fact that humans seem to have used much more complex recognition strategies than the models of current study.

One limitation of current study, which might seem to constrain the generalization of the results to the big problem of object recognition, is the number of objects used in the experiments. This limitation was imposed on the study by two factors: the Bubbles method and the large number of variation conditions needed to study invariant object recognition. When using the Bubbles method, to gain statistical significance for diagnostic and anti-diagnostic regions, each object condition needs to be presented to each subject hundreds of times (i.e. our subjects participated in 10 sessions of psychophysical experiments for only a pair of objects). As we aimed to study the dynamical behavior of object features across variations, we did not limit the variation conditions, but limited the number of objects in the image set. Nonetheless, two is the most common number for objects used with Bubbles method^36,37,62^, and the number has been rarely increased^29,63^. We believe that, although the diagnostic features will surely vary across different sets of objects, the strategies will most probably remain stable across objects. This is supported by many of the human strategies observed in the current study which repeated strategies observed in rats^36,37^; two totally different species discriminating drastically different object categories. However, the inclusion of factors such as levels of categorization (i.e. subordinate, basic and superordinate)^64^ and task complexity (e.g. detection, discrimination, attention)^60^ will probably affect the developed strategies.

Another limitation of this study, which can be covered in the future, is disregarding the effect of cluttered background on the choice of diagnostic features. It has been shown by many studies that object background has drastic impacts on the recognition performance of objects (i.e. also under variations) in humans^4,46,64^, and that background can also be considered diagnostic by humans in complex discrimination tasks^30^. Therefore, it seems relevant to study the effect of background on diagnostic features. These backgrounds can be constructed artificially to provide low-level control (e.g. random noise, scrambled phase images, etc.)^46^ or can be chosen from natural images^2–4^. This is interesting since background, similar to current influences of object similarity on high-level cognitive processes, also involves top-down (e.g. attention and expertise^35,65^) and bottom-up (e.g. figure-ground segregation^4,18^) mechanisms of the brain.

Although the psychophysical experiments of current study allowed us to investigate the dynamical nature of invariant object recognition at behavioral level, the relative contributions of diagnostic features against other task-related cognitive processes which were active during the experiments (e.g. decision making, attention, task-related motor preparation) and may have contributed to the observed responses have remained unknown. In order to evaluate the contribution of the diagnostic features in object recognition, neuroimaging techniques can be adopted in passive object presentation paradigms. The use of methods such as EEG, EMG and fMRI, along with proper decoding schemes such as reverse-correlation^50^ would be suitable choices in that regard. Moreover, methods such as Granger causality can be used to highlight the role of feed-forward and feedback mechanisms of the brain in feature-based object recognition^17^.

In summary, the quantitative investigation of feature-based object recognition in humans performed in this study, while posing many intact questions for future studies, suggests the reliance on view-invariant diagnostic features as a possible strategy which can explain how invariant object recognition is achieved by the human brain, but not by the state-of-the-art hierarchical machine vision algorithms.

## Electronic supplementary material

Supplementary Information
