# Supplementary material for: Invariant object recognition is a personalized selection of invariant features in humans, not simply explained by hierarchical feed-forward vision models
Source: Sci Rep. 2017 Oct 31;7:14402. doi: 10.1038/s41598-017-13756-8 (PMC5663844; doi:10.1038/s41598-017-13756-8)
Supplement: Supplementary file 1 — Supplementary Information [file 41598_2017_13756_MOESM1_ESM.pdf]

Supplementary Information for:

Invariant object recognition is a personalized selection of invariant features in humans, not simply explained by hierarchical feed-forward vision models

Hamid Karimi-Rouzbahani, Nasour Bagheri, Reza Ebrahimpour

Car model / Car part	Car 1	Car 2	Car 3
Floor			
Roof			
Door			
Hood			
Rear fender			
Trunk / Box			
Front fender			
Front wheel			
Windshield			
Rear window			
Rear wheel			
Rear post			
Window			
Rear bumper			N.A.
Grille			
Exhaust			
Front bumper			N.A.
Quarter window			N.A.
Outside mirror			N.A.
Tail lights			N.A.
Front post			
Middle post			N.A.
Headlights			

Supplementary Table S1. Car parts with their corresponding names which are mentioned throghout the paper. The 3D car models used to generate these images are available under a Creative Commons Attribution-ShareAlike 3.0 Unported License (https://creativecommons.org/licenses/by-sa/3.0/) and were freely downloaded from (https://grey.colorado.edu/CompCogNeuro/index.php/CU3D).
